# Mitochondrial haplotypes influence metabolic traits across bovine inter- and intra-species cybrids

**DOI:** 10.1038/s41598-017-04457-3

**Published:** 2017-06-23

**Authors:** Jikun Wang, Hai Xiang, Langqing Liu, Minghua Kong, Tao Yin, Xingbo Zhao

**Affiliations:** National Engineering Laboratory for Animal Breeding; Key Laboratory of Animal Genetics, Breeding and Reproduction, Ministry of Agriculture; College of Animal Science and Technology, China Agricultural University, 100193 Beijing, China

## Abstract

In bovine species, mitochondrial DNA polymorphisms and their correlation to productive or reproductive performances have been widely reported across breeds and individuals. However, experimental evidence of this correlation has never been provided. In order to identify differences among bovine mtDNA haplotypes, transmitochondrial cybrids were generated, with the nucleus from MAC-T cell line, derived from a Holstein dairy cow (*Bos taurus*) and mitochondria from either primary cell line derived from a domestic Chinese native beef Luxi cattle breed or central Asian domestic yak (*Bos grunniens*). Yak primary cells illustrated a stronger metabolic capacity than that of Luxi. However, all yak cybrid parameters illustrated a drop in relative yak mtDNA compared to Luxi mtDNA, in line with a mitonuclear imbalance in yak interspecies cybrid. Luxi has 250 divergent variations relative to the mitogenome of Holsteins. In cybrids there were generally higher rates of oxygen consumption (OCR) and extracellular acidification (ECAR), and lower mRNA expression levels of nuclear-encoded mitochondrial genes, potentially reflecting active energy metabolism and cellular stress resistance. The results demonstrate that functional differences exist between bovine cybrid cells. While cybrid viability was similar between Holstein and Luxi breeds, the mitonuclear mismatch caused a marked metabolic dysfunction in cattle:yak cybrid species.

## Introduction

The mitochondrion is a complex organelle that houses essential pathways involved in energy metabolism, ion homeostasis, signal transduction and apoptosis^1^. Both mitochondrial and nuclear genomes must be highly compatible to maintain the structural and biochemical properties for OXPHOS function^2^. Because of these close interactions, mitochondrial and nuclear genomes undergo adaptive co-evolution to maintain fitness in energy metabolism^3^; thus, interspecies incompatibility of nDNA-mtDNA could represent perturbations to a interspecies generated OXPHOS system^4^.

Particular mtDNA mutants or haplotypes were reported in association with divergent human populations, pathogenesis associated with human disease^5^, performance of elite athletes^6^ and a variety of complex traits^7^. In farm animals, mtDNA variations were also reported to correlate with economic traits, including production, reproduction and stress resistance^8–12^.

The yak is the only native bovine species on the Qinghai-Tibetan Plateau. Yaks readily adapt to the extremely high altitude, and both cold and harsh environments. Yaks are used widely in agricultural practices, providing milk, meat, and fur for local herders^13, 14^; yet, production performance is lower compared to commercial dairy cattle including Holsteins and beef cattle breeds including Luxi (note: Luxi cattle were originally produced for both beef and dairy production; derived from cross breeding *Bos taurus* and *Bos indicus* ^15^. However, the herd used in this study was evaluated by mitogenomic analysis and found to be comprised solely of *Bos taurus* genetics). The cattle-yak is the crossbred of the two species which shows higher heterosis of production traits compared to the yak, but the adaption and male sterility in cattle-yak hybrids provide a biological obstacle to enhancing production characteristics^16^. Additionally, mtDNA divergence and impact on cattle:yak hybrid production traits are unclear.

Because of confounding variations in the nuclear genome, epigenetic phenomena and environmental factors, it was difficult to evaluate the contribution that mtDNA variants might have to complex trait characteristics. Transmitochondrial cybrids (cytoplasmic hybrids) are created by fusing cells devoid of mtDNA (ρ^0^ cells) with cytoplasts (enucleated cells) from different individuals, so the resultant cybrids have uniform nuclear background but different mtDNA. With the development and use of the cybrid (cytoplasmic hybrid) model, questions related to the importance of the mtDNA variants and mitochondrial–nuclear interactions can be addressed^17–19^. In this study, we chose the well-characterized bovine mammary alveolar cell line (MAC-T, from a Holstein cow) as the nucleus donor, and somatic cell lines from a yak (*Bos grunniens*) and a Chinese native cattle, Luxi (*Bos taurus*) as mitochondria donors, to generate bovine intra- and inter-species cybrids. Mitogenome variations and expression measures were evaluated including, oxygen consumption rate (OCR), extracellular acidification rate (ECAR), mtDNA copy number, mitochondrial biogenesis related gene haplotypes, and fat synthesis related gene haplotypes in primary cells and cybrids. This study provides the first evidence detailing mtDNA effects using transmitochondrial cells across intra- and inter-bovine species.

## Results

### Creation of cybrids

MAC-T cells (C0) were treated to generate ρ^0^ cells, while Holstein MAC-T (C0), primary fetal fibroblasts from a female yak (Y) and a Chinese native beef cow (Luxi, C1) were used as mitochondria donors to generate cybrids (C0+C0, C0+Y and C0+C1), respectively. After selection at 5-week of culture, we performed PCR amplified sequencing of mtDNA control regions to confirm the absence of endogenous and the complete substitution of exogenous mtDNA in each cybrid cell. Results showed that C0+Y and C0+C1 contained the Y and C1 mtDNA, respectively, and the endogenous host mtDNAs were no longer detectable (Supplementary Fig. S1).

### Mitogenome sequencing and analyses

The complete mitochondrial genome sequences of the three bovine cell lines (C0, C1 and Y) were determined and deposited in GenBank with accession numbers of KU891849-KU891851. Cybrid cells (C0+C0, C0+C1 and C0+Y) were also sequenced, and the mitogenome sequence of each cybrid cell was consistent with its mitochondria donor.

The mitogenomes generated divergence sequences amongst the three primary cell lines (Table 1); representative of the different bovine species or breeds. Specifically, the alignment C0/C1 presented 250 variations, while C0/Y and C1/Y harbored 1037 and 1010 variations, respectively. For functional analyses, C0/C1 harbored 165 mutations on the 13 mtDNA protein-coding genes, including 20 missense mutations which were all with conservative or moderately conservative Grantham scores (0–50 for conservative, 51–100 for moderately conservative, 101–150 for moderately radical, >150 for radical)^20, 21^. Meanwhile, C0/Y and C1/Y harbored 84 and 58 missense mutations, respectively. Of particular note, C0/Y harbored 2 missense mutations (Ser269Leu in *ND1* and Ser101Leu in *ND4*), which appeared as radical changes with a Grantham score of 145. Detailed mutation data can be found in Supplementary Table S1 and S2.

**Table 1:** Mitogenome divergences across the three primary cells.

Comparison	SNPs/InDels					AA change	Grantham Score summary
	D-loop	rRNA	tRNA	Protein	Total		
C0/C1	47	25	13	165	250	20	12 in 0–50, 8 in 51–100
C0/Y	115	102	45	775	1037	84	38 in 0–50, 44 in 51–100, 2 in 101–150
C1/Y	111	100	44	755	1010	58	28 in 0–50, 30 in 51–100

**Figure 1:**
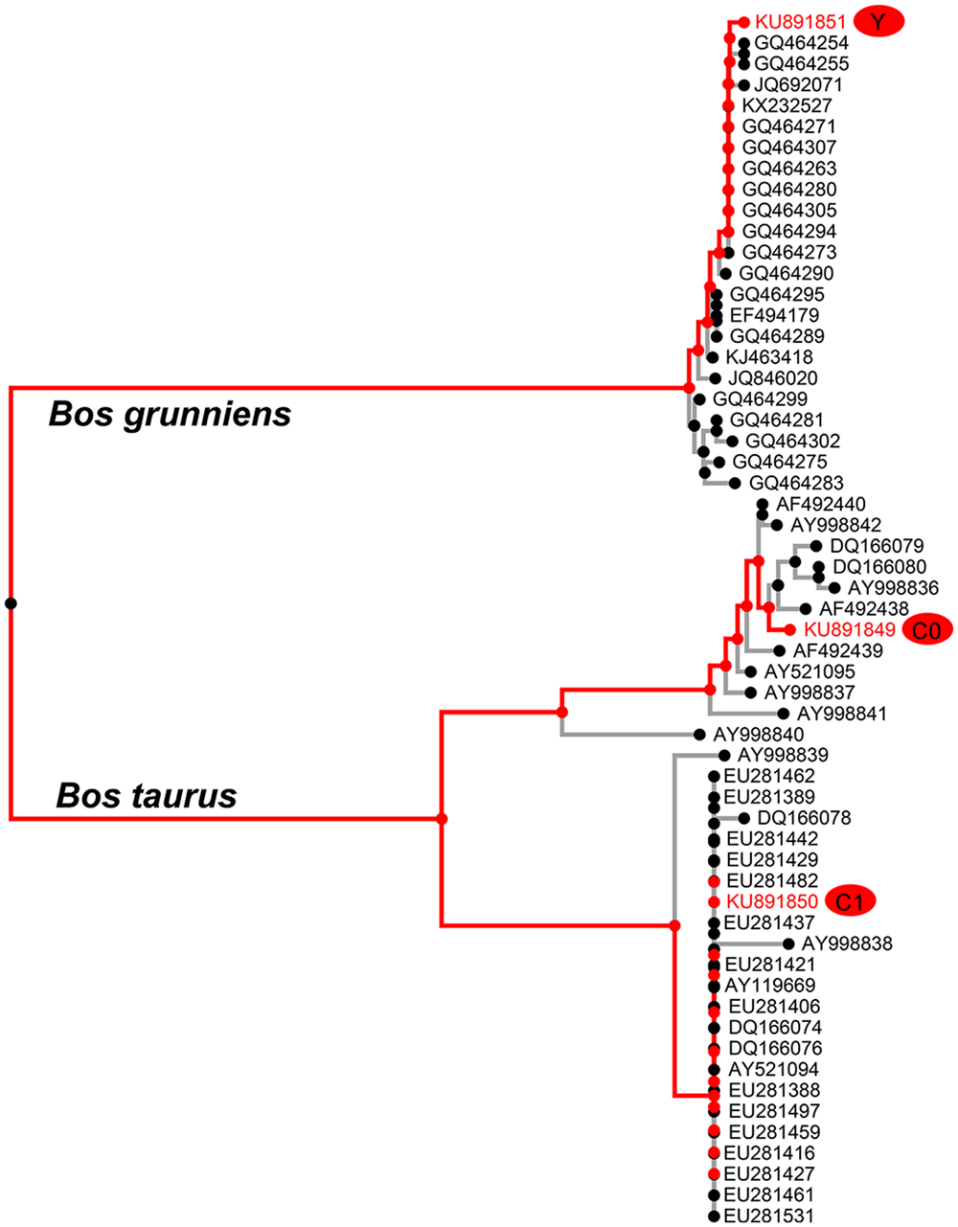
Relationship of the three bovine primary cells of yak, Holstein and Luxi revealed by mitochondrial control sequences. Codes indicating GenBank entries, red codes indicating primary cells of yak (Y), Holstein (C0) and Luxi (C1).

A consensus NJ tree based on control region sequences from Holstein, Luxi and yak females was constructed, which resulted in two apparent clusters (Fig. 1). C0 and C1 were grouped in the clad of *Bos taurus*, precisely, they clustered into different branches. And Y was belonged into *Bos grunniens*.

### Oxygen consumption rate (OCR) and extracellular acidification rate (ECAR) assays

A Seahorse XFe96 Analyzer was used to measure OXPHOS and glycolysis properties of bovine primary and cybrid cells.

**Figure 2:**
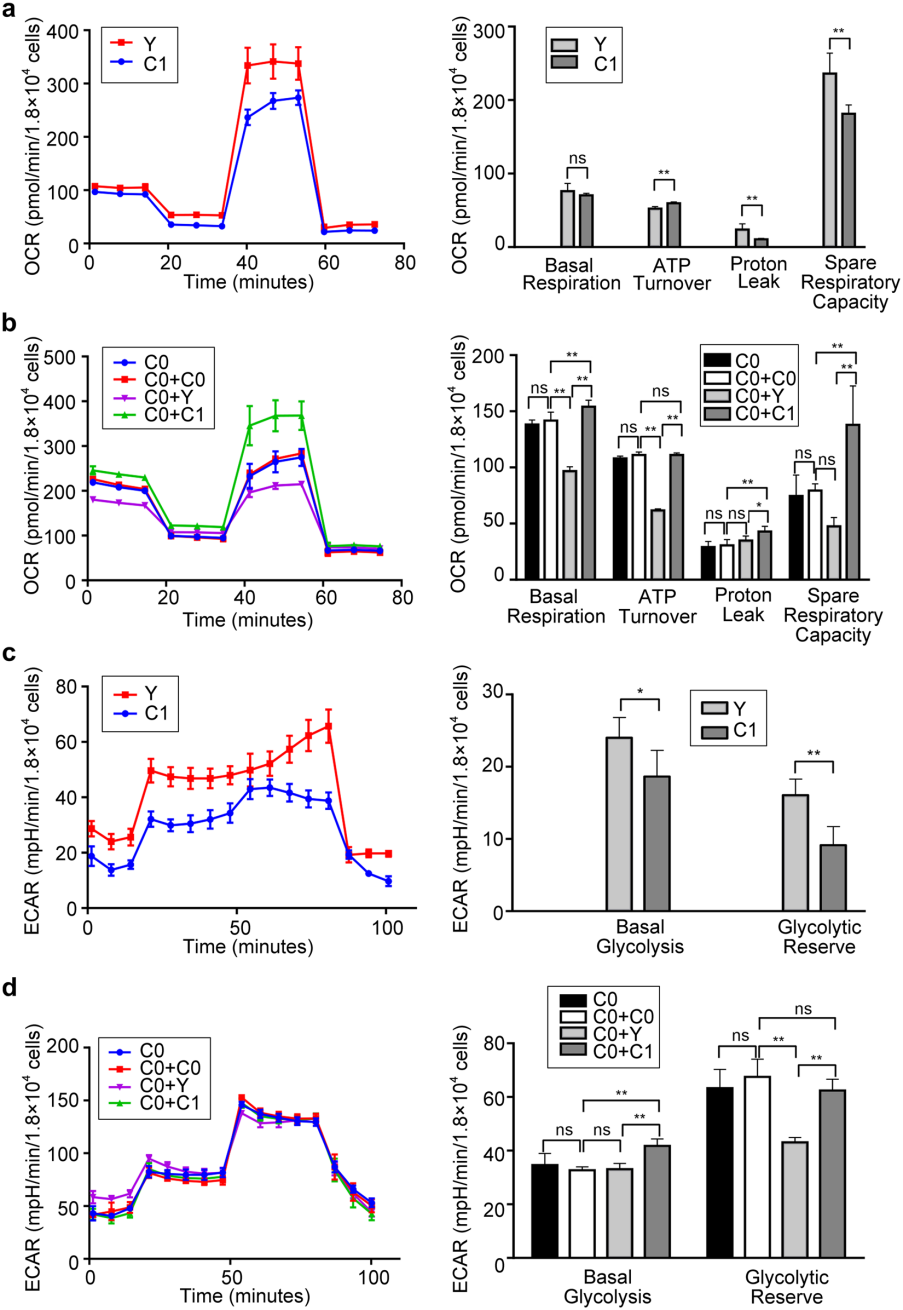
OCR and ECAR assays for bovine primary cells and cybrid cells. **a** and **c** illustrating OCR and ECAR assays of primary cell lines (Y and C1). **b** and **d** illustrating OCR and ECAR assays of cybrid cells (C0+C0, C0+C1, C0+Y), C0 (MAC-T) was used as a control for C0+C0. For OCR assays, all cells exposed sequentially to oligomycin, FCCP and rotenone plus antimycin A. Non-mitochondrial respiration was subtracted from the other values, basal respiration, ATP turnover, proton leak, and spare respiratory capacity. OCR profiles were expressed as pmole O2/min/1.8 × 10^4^ cells. The basal glycolysis rate for each cell line was estimated by determining its ECAR in the presence of glucose. The glycolytic reserve for each cell line was estimated by determining the ECAR in the presence of oligomycin. ECAR profiles were expressed as mpH/min/1.8 × 10^4^ cells. In cybrid cells, the acronym C0 denotes a common nucleus of MAC-T, and +C0, +Y and +C1 represent the source of mitochondria. Bars indicate the standard deviation (SD), *n* = *6* per group. *P < 0.05, **P < 0.01 and ns *P* > 0.05.

For yak (Y) and Luxi (C1) primary cells, Y exhibited statistically higher ECAR indexes than that of C1 (basal glycolysis at *P* < 0.05 and glycolytic reserve at *P* < 0.01, respectively) (Fig. 2c). However, the yak presented similar basal aerobic respiration with Luxi cell line, with which presented significantly lower ATP turnover and higher proton leak (*P* < 0.01) (Fig. 2a), indicating that the yak cell has ordinary capability for aerobic respiration. Meanwhile, higher levels of proton leak and spare respiratory capacity were observed in the yak cell line with evidence of resistance to cold stimulation and respiration stress (*P* < 0.01).

Unlike primary cells, the yak cybrid (C0+Y) presented lower OCRs and ECARs than Luxi cybrid (C0+C1), and lower parameters of basal aerobic respiration, ATP turnover and glycolytic reserve (*P* < 0.01) than the Holstein cybrid (C0+C0) (b and d in Fig. 2), illustrating a perturbation in mitochondrial function and energy metabolism in the yak cybrid. The Luxi cybrid exhibited higher basal respiration (aerobic and anaerobic), and greater proton leak and spare respiratory capacity than the Holstein cybrid (*P* < 0.01) (b and d in Fig. 2), demonstrating a dramatic effect on energy metabolism.

The Seahorse results allowed us to infer metabolic affinities for bovine primary cells and cybrids. For aerobic respiration analyses, an ATP turnover module consisted of all reactions involved in phosphorylation of ADP to ATP and the export and turnover of ATP in the extramitochondrial space^22^. Thus, alteration in mitochondrial ATP concentrations could markedly influence economic traits^23^. Proton leak pathways regulate physiological processes including nonshivering thermogenesis and perhaps glucose-stimulated insulin secretion and protection from oxidative damage^24^. Spare respiratory capacity represents the extra mitochondrial capacity available in a cell to produce energy under conditions of increased work or stress and is thought to be important for long-term cell survival and function^25^. Accordingly, the results outlined here illustrate striking biological effects likely to impact production traits.

### Quantification of mtDNA copy numbers

**Figure 3:**
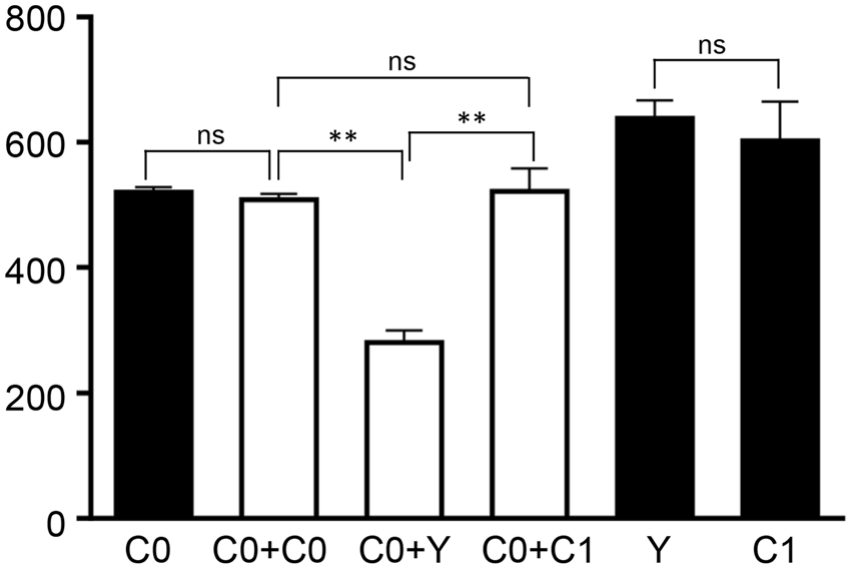
Measurement of mtDNA copy number in primary cells and cybrids. Black columns, primary cells; white columns, cybrid cells. Bars represent standard deviation (SD), *n* = *3* per group. *P < 0.05, **P < 0.01 and ns *P* > 0.05.

mtDNA copy numbers in primary cells and cybrids were measured and presented as the ratio of mitochondrial DNA to nuclear DNA abundance (mtDNA/nDNA) based on Q-PCR assay (Fig. 3). The primary cell yak (Y) and Luxi (C1) were found to carry equal abundance of mtDNA copies. However, within the cybrids, the yak cybrid (C0+Y) harbored lower mtDNA copies than other cybrid cells (*P* < 0.01).

### Expression levels of mitochondrial biogenesis related genes

**Figure 4:**
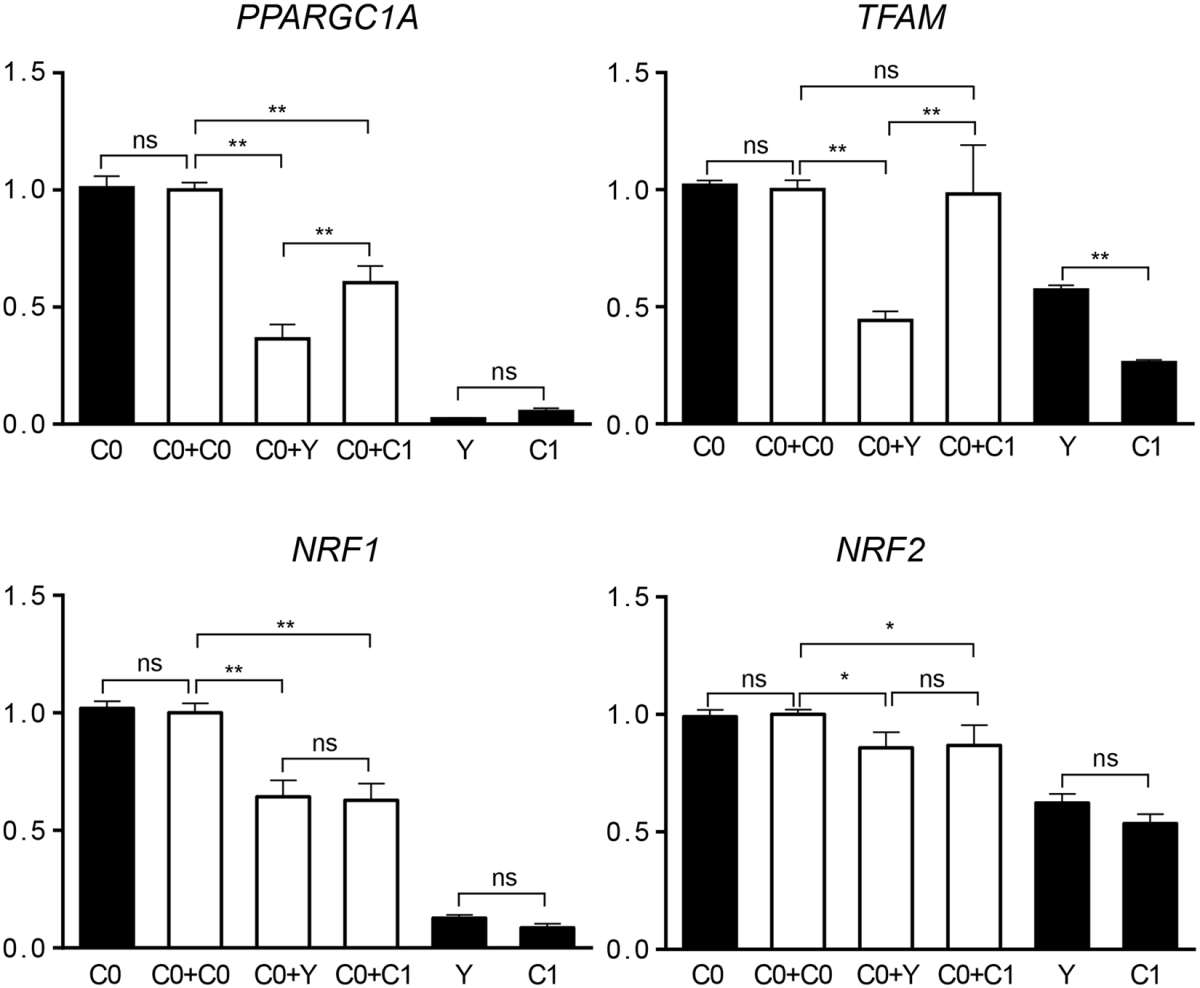
Expression levels of four mitochondrial biogenesis related genes in cybrid cells. Black columns, primary cells; white columns, cybrid cells. Bars indicate the standard deviation (SD), *n* = *3* per group. *P < 0.05, **P < 0.01 and ns *P* > 0.05.

Four mitochondrial biogenesis related genes (*PPARGC1A*, *TFAM*, *NRF1* and *NRF2*) in all cybrid cells were used to measure expression abundance (Fig. 4). C0 and C0+C0 presented consistent expression levels in all tests, and C0+Y had lower expression abundances of *PPARGC1A* and *TFAM* than other cybrid cells (*P* < 0.01). On *PPARGC1A*, C0+C1 illustrated a lower level than C0+C0 (*P* < 0.01). No change was observed between C0+C1 and C0+C0 in *TFAM*. Similar to C0+Y, C0+C1 had a lower expression abundance of *NRF1* and *NRF2* than C0+C0 (*P* < 0.01 and *P* < 0.05, respectively). For yak (Y) and LUXI (C1) primary cells, Y presented higher expression levels of *TFAM* than C1 (*P* < 0.01), and similar level of other three genes evaluated (Fig. 4).

### Expression levels of fat synthesis related genes

**Figure 5:**
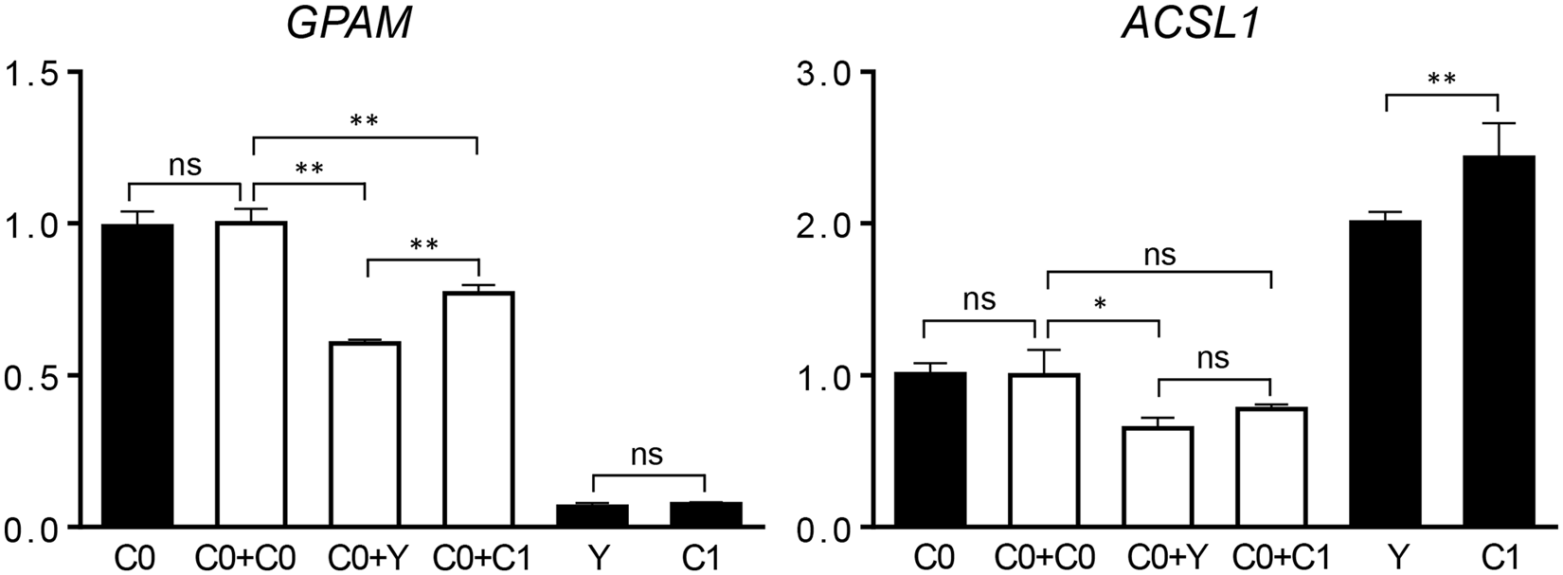
Expression levels of two fat synthesis related genes. Black columns, primary cells; white columns, cybrid cells. Bars indicate the standard deviation (SD), *n* = *3* per group. *P < 0.05, **P < 0.01 and ns *P* > 0.05.

Similar to those of mitochondrial biogenesis related genes, C0 and C0+C0 presented consistent expression levels in all analyses. C0+C1 had higher levels of *GPAM* than C0+Y (*P* < 0.01), but lower levels than C0+C0 (*P* < 0.01). On *ACSL1*, C0+C0 exhibited higher expression abundance than C0+Y (*P* < 0.05) (Fig. 5). For Y and C1 primary cells, Y had lower levels of *ACSL1* expression than C1 (*P* < 0.01), but *GPAM* did not differ between Y and C1.

## Discussion

Dynamic properties associated with energy metabolism in yak primary cell culture were comparable to living yaks. Comparing to native beef cattle (C1) and based on the similar mtDNA copy number (Fig. 3), the yak primary cell (Y) detected higher spare respiratory capacity and proton leak, similar basal respiration, and lower ATP turnover, which allow us to infer a biological adaption to harsh conditions (environmental stressors) and resistance to the extreme cold weather, but coupled with the poor productivity traits (Fig. 2a). Yet, yak cells exhibited higher ECAR indexes (basal glycolysis and glycolytic reserve**)** (Fig. 2c), indicative of an adaption to hypoxic circumstance.

The yak is quite phylogenetically different from domesticated cattle (*Bos taurus*) based on mitogenome sequence of both dairy cattle or native beef cattle (Table 1 and Fig. 1), which is also reflected of production and adaptive performance measures of the studied species.

Compared to cattle cybrids (C0+C0 and C0+C1), the yak cybrid (C0+Y) manifested generally lower levels of functional properties, including OCR and ECAR indexes (b and d in Fig. 2), lower mtDNA copy numbers (Fig. 3), mRNA expressions of mitochondrial biogenesis related genes (Fig. 4) and fat synthesis related genes (Fig. 5). OCR and ECAR analysis, examining the performance of the particular mitochondria carriers, illustrated that yak primary cell mitochondria behaved “stronger” than those of Luxi cells (a and c in Fig. 2). Nonetheless, yak data appeared “weaker” in cybrid cells, which manifested prominently lower OCR and ECAR scores (b and d in Fig. 2). These results support the hypothesis of mitonuclear incompatibility in cattle-yak hybrid lines reflecting biological differences that would lead to production trait-related deficiencies; commonly recognized in a variety of interspecies hybrids^13, 26, 27^. A broad range of interactions occur between genomes, including not only those between protein subunits in enzyme complexes, but also protein/RNA and protein/DNA interactions required for the replication, transcription, and translation of organellar genomes^28^. Interactions between mitochondrial genes and their nuclear-encoded genes may be targets of compensatory molecular evolution, and the mitochondrial–nuclear interaction provides a plausible mechanism to explain this complexity^29^.

Luxi cattle are a native beef breed in China, which through natural selection and subsequent husbandry practices, adapt extremely well to endemic environments. However, its dairy performance is considerably lower than the European-derived commercial Holstein breed. Compared to Holstein control lines (C0+C0), Luxi cybrid (C0+C1) exhibited generally higher OCR and ECAR, with an increase in basal respiration, spare respiratory capacity, basal glycolysis and proton leak (b and d in Fig. 2), reflecting an active energy metabolism.

*PPARGC1A*, *TFAM*, *NRF1* and *NRF2* are nuclear-encoded mitochondrial genes, which regulate mitochondrial biogenesis and metabolic processes^30^. *GPAM* and *ACSL1* are fat synthesis related genes during bovine mammary tissue and their expression is affected by stage of lactation^31, 32^. In this study, based on parallel amount of mtDNA copy numbers between Luxi and Holstein cybrids (Fig. 3), Luxi cybrid harbored lower mRNA expression levels for nuclear-encoded mitochondrial genes (Fig. 4) and fat synthesis-related genes (Fig. 5), and ordinary level of ATP turnover (Fig. 2b), illustrating the mediocre ATP supply for production coupled with a poor milk output.

Representative mitochondrial haplotypes of Luxi (C1) and the Holstein cows (C0), presented 250 SNPs differences (Table 1 and Fig. 1). The results that Luxi and Holstein cybrid models produced differences on energy metabolic traits and gene expression levels evidenced mitochondrial haplotype effects on cellular characters and potential tendency of dairy related genes.

In conclusion, this is the first study, to our knowledge, that demonstrates specific mitochondrial haplotypes, which ultimately confer specific functional differences in mitochondrial metabolism using transmitochondrial cells of inter- and intra-bovine species. This study provides insight in characterizing mtDNA haplotypes and subsequent biological sequelae that effectively impact metabolic traits of domestic animals.

## Methods

### Ethics statement

The guidelines of the experimental animal management of China Agricultural University (CAU) were followed throughout the study, and the experimental protocols were approved by the Experimental Animal Care and Use Committee of CAU.

### Cell lines and culture conditions

The bovine mammary alveolar cell line (MAC-T, C0) from a Holstein cow (*Bos taurus*) in this study was presented by Dr. Zhao Fengqi, University of Vermont, Burlington, USA. Primary fetal fibroblasts were isolated from two cows: C1 from the Luxi cattle breed (*Bos taurus* independently confirmed by mitogenome sequence analysis in this study), and Y from the Datong yak breed (*Bos grunniens*). All cells were cultured in DMEM (Gibco) supplemented with 10% FBS (Gibco), 100 units/ml penicillin and 100 μg /ml streptomycin (Gibco), 50 μg/ml uridine (Amresco), and 1 mM pyruvate (Sigma) at 37 °C and 5% CO2/ 95% air unless otherwise specified.

### Production of transmitochondrial cybrids

Cybrids were produced by enucleation of mitochondria donor cells and fusion of the cytoplasts with ρ^0^ cells according to modified procedures of Bacman and Moraes^33^. Plasmid pSV2-neo has been transfected into MAC-T cells (C0) to resist to G418 as a selective marker^34^. C0 was used as nucleus donor (ρ^0^ cell), which was treated with 3 μg/ml rhodamine 6G (R-6G) in medium for 6 d with replacement of medium at 24-h intervals. ρ^0^ cells were cultured in normal medium for 3 h before cytoplast fusion. C0, C1 and Y cells were used as mitochondria donors to generate the cybrids C0+C0, C0+C1 and C0+Y, respectively.

### Mitogenome sequencing

Totally, 18 pairs of PCR primers were designed to generate overlapping fragments covering bovine mitochondrial genome (Supplementary Table S3). Sequences were inspected and assembled using DNASTAR Lasergene 10.1 software (http://www.dnastar.com/) and deposited at GenBank. Then SNPs among these three sequences were found out, and missense mutations were established the Grantham Score matrix referring to the amino acid difference formula^20, 21^.

Mitochondrial DNA control region sequences generated in this study and corresponding sequences for Holstein, Luxi cattle, yak and other Chinese native bovine breeds were downloaded from GenBank. Sequence alignment was performed using online MAFFT software^35^ with default parameters. Then all gap-free sites were applied to constructing consensus NJ tree with Jukes-Cantor model and 1000 bootstraps by the online MAFFT software^35^, and the consensus tree was depicted using the web application Phylo.io^36^.

### Respiration and glycolysis analyses

Approximately 1.8 × 10^4^ cells for each cell line were seeded in 6 wells of XF96 cell culture microplates (Seahorse Bioscience). For respiratory analyses, cells were analyzed according to the procedures described in the Seahorse XF Cell Mito Stress Test kit. After baseline measurements of OCR, OCR was measured after sequentially adding to each well Oligomycin (1.5 μM final concentration, which inhibit ATP synthase to measure respiration required for ATP turnover), FCCP (carbonyl cyanide 4-trifluoromethoxy-phenylhydrazone, 0.5 μM final concentration, a protonophoric uncoupler which induces maximal respiration), and Rotenone plus Antimycin A (0.5 μM final concentration of each, which completely inhibits the mitochondrial respiratory chain to measure non-mitochondrial OCR contribution). Subtracting the non-mitochondrial OCR from the total OCR yields the mitochondrial OCR. For glycolysis analyses, cells were analyzed according to the procedures described in the Seahorse Glycolysis Stress Test kit. Briefly, initial measurements taken in the absence of glucose, and again after an injection of 2-deoxyglucose (to a final concentration of 100 mM), generate a non-glycolysis extracellular acidification rate (ECAR). In between these two baseline ECAR measurements, glucose was added to each well at a concentration of 10 mM. The resulting ECAR minus the non-glycolysis ECAR yielded the glycolysis ECAR. Next, oligomycin was added to each well (at a 1.5 μM concentration). The resulting ECAR minus the non-glycolysis ECAR provided the glycolysis capacity ECAR. Subtracting the glycolysis ECAR from the glycolysis capacity ECAR provided the glycolysis spare reserve capacity. Data were normalized by cell number (1.8 × 10^4^ cells) measured by the CyQUANT Cell proliferation kit (Invitrogen). OCRs were expressed as pmol O2/min/1.8 × 10^4^ cells, ECARs were expressed as mpH/min/1.8 × 10^4^ cells. Experiments were repeated three times. All data from XFe96 assays were collected using the XF reader software from Seahorse Bioscience.

### Quantification of mtDNA copy number

We followed the DNA extraction method suggested by Guo *et al.* ^37^ to avoid underestimating the abundance of mitochondrial DNA. The mtDNA specific primers (F: 5′-AATCCTACAAATCCTCACAGG-3′, positions at 14637-14657; R: 5′-TTGAAGCTCCGTTTGCGTGT-3′, positions at 14760-14779) were designed on the basis of the GenBank nucleotide sequence (KF926377.1). The *GAPDH* gene was used as the internal standard (F: 5′-GTGATGCTGGTGCTGAGTAT-3′ and R: 5′-GCTCTCACATTCCTAAGTCC-3′). The product lengths were 139 bp (mtDNA fragment) and 143 bp (GAPDH fragment) respectively. The mtDNA copy number of each sample was compared by calculating the ratio of mitochondrial to nuclear DNA abundance (mtDNA/nDNA). Each Q-PCR experiment was performed in triplicates.

### The expression levels of mitochondrial biogenesis related genes and fat synthesis related genes

In the present study, the expression levels of 4 mitochondrial biogenesis related genes (*PPARGC1A*, *TFAM*, *NRF1 and NRF2*) and 2 fat synthesis related genes (*GPAM* and *ACSL1*) were used to evaluate the metabolic differences amongst cybrids. We aimed to focus on the differences between C0+C1 and C0+Y, however, the primary cell MAC-T and cybrid C0+C0 were also investigated as controls, and the expression abundance of C0+C0 was set as 1.

Total RNA were isolated using RNeasy Mini-Extraction kit (Qiagen) and quantified using M200 Pro Nanoquant (Tecan). Then 100 ng of each RNA sample was reverse transcribed using the QuantiTect Reverse Transcription Kit (Qiagen) for Q-PCR analyses. *GAPDH* was used as a reference gene for qPCR experiments, and all primers were successfully used in bovine^38–41^. The primer sequences and information are listed in Supplementary Table S4. The Q-PCRs were performed using Bio-Rad iCycler iQ5 detection system. Each detection was performed 3 technical replicates and set along with 3 biological replicates.

### Statistical analyses

Data was subjected to statistical analysis by ANOVA. Tukey’s multiple comparison test was done to compare the data within each experiment. Error bars in the graphs represent SD (standard deviation).

## Electronic supplementary material

Supplementary materials
