# Supplementary material for: Mitochondrial haplotypes influence metabolic traits across bovine inter- and intra-species cybrids
Source: Sci Rep. 2017 Jun 23;7:4179. doi: 10.1038/s41598-017-04457-3 (PMC5482896; doi:10.1038/s41598-017-04457-3)
Supplement: Supplementary file 1 — Supplementary materials [file 41598_2017_4457_MOESM1_ESM.pdf]

Supplementary materials for

Mitochondrial haplotypes influence metabolic traits across bovine inter- and intra-species cybrids

Jikun Wang[1], Hai Xiang[1], Langqing Liu[1], Minghua Kong[1], Tao Yin[1]& Xingbo Zhao[1,*]

[1] National Engineering Laboratory for Animal Breeding; Ministry of Agriculture, Key Laboratory of Animal Genetics, Breeding and Reproduction; College of Animal Science and Technology, China Agricultural University, 100193, Beijing, China.

[*]To whom correspondence may be addressed. E-mail: zhxb@cau.edu.cn, Tel: +86-010-62733417, Fax: +86-010-62733417.

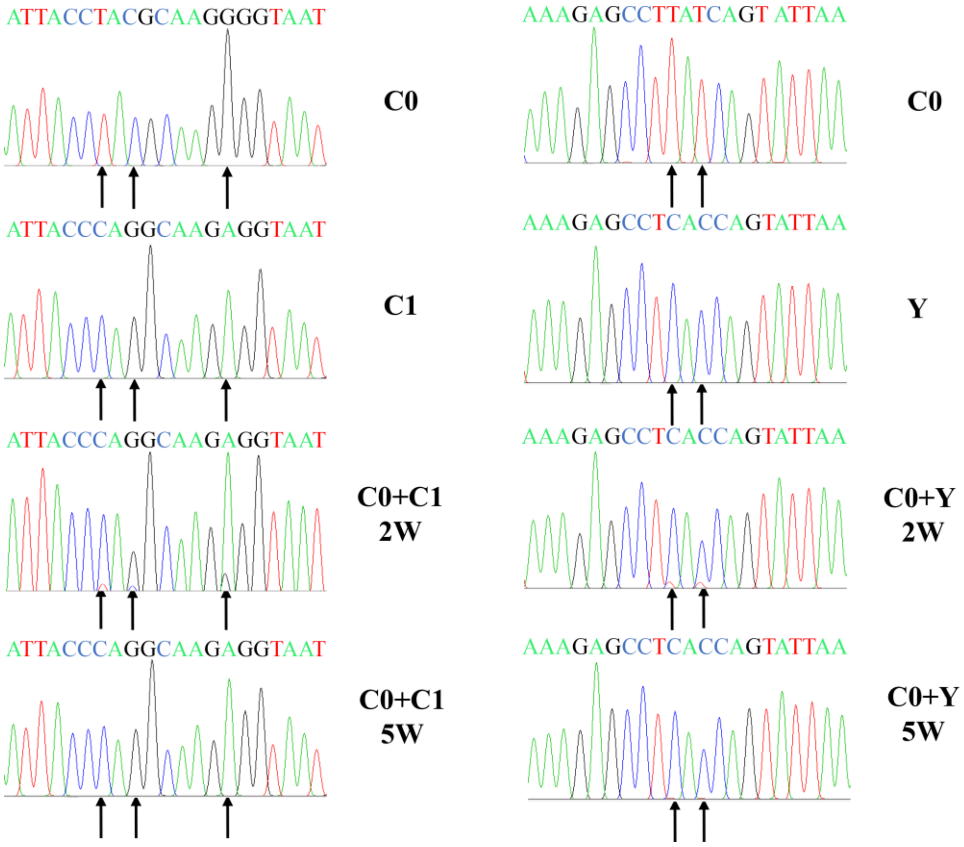

Fig. S1 Sequence verification in the generation of different cybrid cells. C0, C1 and Y exhibit characteristic sequence signatures within the D-loop region. All cybrids at the second week (C0+C1/2W and C0+Y/2W) were characterized with double peaks, indicating the coexistence of endogenous and exogenous mitochondria (containing mtDNAs). The endogenous mtDNAs gradually degraded and were no longer detectable at the fifth week (C0+C1/5W and C0+Y/5W). In cybrids, the acronym C0 denotes a common nucleus of MAC-T, and +C0, +Y and +C1 represent the source of mitochondria. 2W, 5W: cell culture for 2 weeks and 5 weeks, respectively. Arrows indicated specific mutations for a particular mtDNA haplotype.

Table S1 Detailed variant sites among MAC-T, Luxi, yak mitogenomes

		1	1	1	1	1	1	1	2	2	2	2	2	2	2	2	2	2	2	2	2	2	3	3	3	3	3	3	4	4	5	5	5
		0	6	6	7	7	7	7	0	0	1	2	3	3	4	5	5	6	8	8	9	9	0	1	2	3	8	8	1	5	1	2	2
	8	6	6	9	2	3	6	9	1	6	6	4	3	4	9	0	6	9	0	2	3	7	1	6	8	0	0	1	9	9	9	6	7
KF926377	G	T	A	G	G	A	T	C	A	T	C	T	T	T	C	T	C	C	A	A	T	T	G	T	A	C	C	C	A	A	A	C	T
MAC-T	.	.	.	.	.	.	.	.	.	.	.	.	.	.	.	.	.	.	.	.	.	.	.	.	.	.	.	.	.	.	.	.	.
Luxi	A	C	G	A	.	G	.	.	.	C	-	.	C	C	T	C	.	.	.	.	.	C	A	.	.	.	.	.	.	.	G	.	.
yak	.	C	G	A	C	.	-	T	G	.	.	C	-	.	T	.	T	T	T	G	A	C	A	A	T	T	T	T	G	G	G	T	C

																			1	1	1	1	1	1	1	1	1	1	1	1	1	1	1
	5	6	7	7	7	7	7	7	7	7	7	8	8	8	9	9	9	9	0	0	0	0	1	1	1	1	1	1	2	2	3	3	4
	5	5	1	2	2	3	4	4	5	6	6	1	1	2	1	2	7	8	1	8	8	8	3	6	6	9	9	9	2	9	1	3	5
	6	1	1	4	8	9	1	2	8	2	3	8	9	6	1	5	0	9	9	4	5	9	4	0	8	2	3	5	8	8	0	0	9
KF926377	T	T	G	C	T	T	C	C	C	C	T	A	T	C	C	T	T	A	A	G	T	T	G	C	T	C	C	A	A	G	C	C	G
MAC-T	.	.	.	.	.	.	.	.	.	.	.	.	.	.	.	.	.	.	.	.	.	.	.	.	.	.	.	.	.	.	.	.	.
Luxi	.	.	.	T	.	C	.	.	.	.	C	G	.	.	.	.	.	.	.	.	.	.	.	T	.	.	.	.	.	.	.	.	.
yak	C	C	A	.	C	C	T	T	T	T	.	.	C	T	T	C	C	G	G	A	C	C	A	T	C	T	T	G	G	A	T	T	A

	1	1	1	1	1	1	1	1	1	1	1	1	1	1	1	1	1	1	1	1	1	1	1	1	1	1	1	1	1	1	2	2	2
	4	4	4	4	4	4	4	4	5	5	5	5	6	6	6	6	7	7	7	8	8	8	8	8	8	8	8	9	9	9	0	0	0
	6	7	7	8	8	9	9	9	0	5	5	5	1	7	8	9	1	1	3	2	2	2	3	6	7	7	8	4	8	8	1	2	2
	1	2	6	2	3	1	4	7	1	4	5	7	1	8	5	5	7	9	0	3	4	5	4	1	0	2	0	9	2	8	7	2	7
KF926377	C	G	C	C	G	A	C	G	G	G	T	C	T	T	T	G	C	C	C	A	A	A	A	T	C	C	G	T	T	A	T	T	T
MAC-T	.	.	.	.	.	.	.	.	.	.	.	.	.	A	.	.	.	.	.	.	.	.	.	.	.	.	.	.	.	.	.	.	.
Luxi	.	.	T	.	.	.	T	.	A	.	.	.	.	C	.	.	.	.	T	.	.	G	.	A	T	.	.	.	.	.	C	.	.
yak	T	A	.	T	A	T	.	A	A	A	C	T	C	.	C	A	T	T	T	G	T	.	T	.	T	T	A	C	C	G	C	C	C

	2	2	2	2	2	2	2	2	2	2	2	2	2	2	2	2	2	2	2	2	2	2	2	2	2	2	2	2	2	2	2	2	2
	0	0	0	0	0	1	1	1	1	1	1	1	2	2	2	3	3	3	4	5	5	5	5	5	5	6	6	7	7	8	9	9	9
	3	3	4	7	8	0	1	4	6	7	7	8	0	0	2	1	2	3	3	0	5	5	7	7	7	3	4	0	4	7	5	6	6
	2	8	0	8	8	0	8	6	6	3	4	6	7	9	1	9	2	6	5	5	4	9	1	6	7	5	3	1	9	8	4	6	8
KF926377	C	A	G	A	C	T	T	G	C	T	C	T	G	T	C	T	C	T	G	C	G	G	A	A	T	T	G	C	C	T	C	G	C
MAC-T	.	.	.	.	.	.	.	.	.	.	.	.	.	.	.	.	.	.	.	.	.	.	.	.	.	.	.	.	.	.	.	.	.
Luxi	.	.	.	.	.	C	.	.	.	.	.	.	.	.	.	.	.	.	.	.	.	A	.	G	.	C	.	.	.	.	T	.	.
yak	T	G	A	T	T	.	C	A	T	C	T	C	A	C	T	C	T	C	A	T	A	A	G	.	C	.	A	T	T	C	T	A	T

	2	2	2	2	2	2	2	3	3	3	3	3	3	3	3	3	3	3	3	3	3	3	3	3	3	3	3	3	3	3	3	3	3
	9	9	9	9	9	9	9	0	0	1	1	1	1	1	1	1	1	1	1	2	2	2	2	2	2	2	2	3	3	3	3	3	3
	7	7	8	8	8	9	9	5	7	2	3	3	4	4	6	6	7	8	9	2	3	4	7	8	9	9	9	0	1	2	2	2	3
	2	8	0	2	9	0	1	2	2	8	1	7	1	6	1	4	0	7	1	4	9	2	2	2	0	3	6	3	4	3	6	9	0
KF926377	C	T	A	A	C	T	G	A	T	A	T	C	C	G	T	G	G	G	T	T	T	A	T	C	C	T	A	G	T	A	T	C	A
MAC-T	.	.	.	.	.	.	.	.	.	.	.	.	.	.	.	.	.	.	.	.	.	.	.	.	.	.	.	.	.	.	.	.	.
Luxi	.	C	G	.	T	C	A	G	C	.	.	T	.	A	.	.	.	C	.	.	.	G	.	.	.	.	.	.	.	.	C	.	.
yak	T	C	G	G	T	.	A	G	C	T	C	T	T	A	C	A	A	.	C	C	C	.	C	T	T	C	G	A	C	G	C	T	T

	3	3	3	3	3	3	3	3	3	3	3	3	3	3	3	3	3	3	3	3	3	3	3	3	3	3	3	3	3	3	3	3	3
	3	3	3	3	3	4	4	4	5	5	5	5	5	6	6	6	6	6	6	6	6	7	8	8	8	8	8	8	8	8	8	8	8
	3	7	8	8	8	2	4	4	2	3	5	5	7	0	1	2	3	6	9	9	9	9	0	0	2	2	3	3	4	7	8	8	9
	6	8	0	3	9	2	0	9	7	6	1	2	5	1	6	1	8	9	2	8	9	4	6	9	2	7	0	3	5	5	0	4	3

KF926377	T	T	T	T	C	C	A	T	C	G	G	T	G	C	T	T	G	C	A	T	T	T	T	C	T	C	A	A	T	T	C	T	A
MAC-T	.	.	.	.	.	.	.	.	.	.	.	.	.	.	.	.	.	.	.	.	.	.	.	.	.	.	.	.	.	.	.	.	.
Luxi	C	.	C	.	.	.	G	.	.	A	A	.	.	T	.	.	.	.	.	.	.	.	C	.	.	.	.	G	.	.	C	.	.
yak	C	C	.	C	T	A	G	C	T	A	A	C	A	T	C	C	A	T	C	C	C	C	C	A	C	T	.	G	C	C	T	C	G

	3	3	3	3	3	3	3	3	3	3	3	3	4	4	4	4	4	4	4	4	4	4	4	4	4	4	4	4	4	4	4	4	4
	8	9	9	9	9	9	9	9	9	9	9	9	0	0	0	0	0	0	1	1	1	1	2	2	2	2	2	2	2	3	3	3	3
	9	0	0	3	4	5	6	7	8	8	8	8	0	0	1	2	5	7	0	3	4	6	4	5	8	8	8	9	9	1	2	6	6
	6	7	8	2	1	6	6	7	0	3	6	9	1	7	9	2	9	2	9	0	0	9	9	3	6	7	9	4	7	7	9	2	5
KF926377	G	C	C	C	T	A	C	T	T	A	T	G	G	G	C	A	A	C	C	C	C	T	T	T	T	T	T	C	C	T	A	G	T
MAC-T	.	.	.	.	.	.	.	.	.	.	.	.	.	.	.	.	G	.	.	.	.	.	.	.	.	.	.	.	.	.	.	.	.
Luxi	.	.	.	T	.	.	T	C	.	.	C	.	A	.	.	.	G	.	.	.	.	.	.	.	.	.	.	.	.	.	G	.	.
yak	A	T	A	T	C	G	.	.	C	G	C	A	A	A	A	G	G	A	T	T	T	C	C	C	C	C	C	T	T	C	.	A	C

	4	4	4	4	4	4	4	4	4	4	4	4	4	4	4	4	4	4	4	4	4	4	4	4	4	4	4	4	4	4	4	4	4
	3	3	4	4	4	5	5	5	5	6	6	6	6	6	7	7	7	7	7	8	8	8	8	8	8	8	8	8	8	8	9	9	9
	8	9	0	4	4	3	5	6	9	2	2	3	4	5	3	3	4	4	7	2	2	5	6	6	7	7	8	9	9	9	0	1	3
	6	2	7	3	9	1	1	3	3	3	6	5	8	0	1	4	1	4	0	4	7	4	0	3	2	9	4	0	3	5	8	4	8
KF926377	C	C	T	T	G	C	A	G	C	A	G	C	C	T	A	C	G	T	G	T	A	T	C	C	G	T	T	T	C	T	C	T	C
MAC-T	.	.	.	.	.	.	.	.	.	.	.	.	.	.	.	.	.	.	.	.	.	.	.	.	.	.	.	.	.	.	.	.	.
Luxi	.	.	.	C	.	.	.	A	.	.	.	.	.	.	G	T	.	.	A	.	.	.	.	.	A	.	.	.	.	.	.	.	T
yak	T	T	C	.	A	T	T	A	T	G	A	T	T	A	G	.	A	C	A	C	G	C	T	T	A	C	C	C	T	C	T	C	.

	4	4	4	4	4	5	5	5	5	5	5	5	5	5	5	5	5	5	5	5	5	5	5	5	5	5	5	5	5	5	5	5	5
	9	9	9	9	9	0	0	0	0	0	0	0	1	1	1	1	1	1	2	2	2	2	2	2	2	2	2	3	4	5	5	5	5
	4	5	8	9	9	0	4	4	7	8	8	9	0	0	5	6	8	8	2	3	6	6	7	8	8	8	9	0	9	0	0	0	3
	4	0	9	0	8	1	0	6	3	5	8	7	3	6	7	7	1	7	6	8	0	8	3	0	6	9	3	1	4	1	2	7	2
KF926377	T	G	C	G	T	T	T	T	G	T	C	T	C	T	G	A	T	C	G	T	T	C	T	A	G	A	C	G	C	G	C	T	C
MAC-T	.	.	.	.	.	.	.	.	.	.	.	.	.	.	.	.	.	.	.	.	.	.	.	.	.	.	.	.	.	.	.	.	.
Luxi	.	.	A	.	.	.	.	.	.	.	.	.	.	.	.	.	.	.	.	.	.	.	.	.	A	.	.	.	.	.	T	.	T
yak	C	A	.	A	C	C	A	C	A	C	T	C	T	C	A	G	C	T	A	C	C	T	C	G	A	G	T	A	T	A	T	A	.

	5	5	5	5	5	5	5	5	5	5	5	5	5	5	5	5	5	5	5	5	5	6	6	6	6	6	6	6	6	6	6	6	6
	6	6	6	6	7	7	7	7	7	8	8	9	9	9	9	9	9	9	9	9	9	0	0	0	1	1	1	1	2	2	2	2	2
	0	1	3	7	3	4	5	5	8	2	9	0	0	1	3	5	6	6	8	8	9	1	2	5	1	1	2	5	2	3	3	3	5
	5	5	2	1	2	4	6	9	4	5	1	0	6	8	9	1	3	9	1	4	9	4	0	0	0	6	8	8	1	0	3	6	7
KF926377	T	G	A	G	T	T	T	T	C	T	C	A	C	A	C	T	A	T	T	T	C	C	A	G	T	A	T	A	A	C	T	G	T
MAC-T	.	.	.	.	.	.	.	.	.	.	.	.	.	.	.	.	.	.	.	.	.	.	.	.	.	.	.	.	.	.	.	.	.
Luxi	.	A	.	.	.	C	.	.	T	.	T	.	.	G	.	.	.	.	.	.	T	.	.	.	.	T	.	.	.	.	.	A	.
yak	C	A	G	A	C	.	A	C	.	C	T	G	T	.	T	C	G	C	C	C	.	T	G	A	C	.	C	T	G	T	C	A	C

	6	6	6	6	6	6	6	6	6	6	6	6	6	6	6	6	6	6	6	6	6	6	6	6	6	6	6	6	6	6	6	6	6
	2	2	2	2	3	3	3	3	3	3	3	3	3	4	4	4	4	4	4	5	5	5	5	5	5	5	6	6	6	6	6	6	7
	7	7	8	9	0	1	1	4	5	6	7	7	8	0	2	3	5	5	6	0	0	1	3	5	6	6	0	1	3	4	5	5	2
	0	5	1	1	5	4	5	1	3	8	1	5	0	4	8	7	2	8	1	0	9	2	4	4	6	9	8	1	8	7	7	9	8
KF926377	C	A	G	T	C	A	T	C	G	C	T	C	T	A	C	G	T	C	G	G	T	G	C	C	C	T	A	C	G	C	T	G	A
MAC-T	.	.	.	.	.	.	.	.	.	.	.	.	.	.	.	.	.	.	.	.	.	.	.	.	.	.	.	.	.	.	.	.	.
Luxi	.	.	.	.	.	.	.	T	.	T	.	.	C	.	.	A	.	.	A	.	.	.	.	.	.	.	.	.	.	.	.	.	G
yak	T	C	A	C	T	G	C	.	A	.	C	T	C	G	T	A	C	T	A	A	C	A	T	T	T	A	G	T	A	T	C	A	.

	6	6	6	6	6	6	6	6	6	6	6	6	6	6	7	7	7	7	7	7	7	7	7	7	7	7	7	7	7	7	7	7	7
	7	7	7	7	7	8	8	8	8	8	9	9	9	9	0	0	0	0	0	0	1	1	1	1	2	2	3	3	3	3	3	3	3
	4	5	6	7	9	0	1	7	8	9	0	2	3	5	1	2	4	5	7	8	1	3	3	4	2	7	0	3	5	5	6	6	6
	9	9	4	3	4	0	5	8	2	6	5	3	8	6	6	8	4	5	0	2	5	3	6	6	0	7	5	1	7	9	0	2	8
KF926377	T	T	C	C	C	C	T	C	C	T	C	C	A	A	C	C	A	A	C	T	T	C	G	T	T	T	C	T	A	G	T	C	A
MAC-T	.	.	.	.	.	.	.	.	.	.	.	.	.	.	.	.	.	.	.	.	.	.	.	.	.	.	.	.	.	.	.	.	.
Luxi	.	.	.	T	.	.	.	.	T	.	.	T	.	.	.	.	.	.	.	.	.	.	.	.	.	.	T	C	G	A	.	T	.
yak	C	C	T	T	T	T	C	T	.	C	T	.	G	G	T	T	G	G	T	C	C	T	A	C	C	C	.	C	.	A	C	.	G

	7	7	7	7	7	7	7	7	7	7	7	7	7	7	7	7	7	7	7	7	7	7	7	7	7	7	7	7	7	7	7	7	7
	3	4	4	4	5	5	5	5	5	5	5	5	5	5	5	6	6	6	6	6	6	7	7	7	8	8	8	8	8	8	8	8	8
	9	1	1	4	0	1	1	2	2	2	3	6	7	8	8	9	4	5	6	6	7	9	0	7	7	0	3	3	4	5	6	6	7
	8	0	9	9	0	0	5	1	4	7	9	6	6	4	7	0	4	6	2	9	1	8	1	0	1	6	0	1	8	2	6	9	2
KF926377	A	A	A	C	T	C	G	G	G	C	G	C	C	C	C	C	C	T	A	A	C	T	G	G	C	A	G	T	C	T	T	G	C
MAC-T	.	.	.	.	.	.	.	.	.	.	.	.	.	.	.	.	.	.	.	.	.	.	.	.	.	.	.	.	.	.	.	.	.
Luxi	.	.	.	.	.	.	A	.	.	.	.	.	.	.	.	.	.	.	.	.	.	.	.	.	.	.	.	C	.	C	.	.	.
yak	C	T	G	T	C	T	A	A	A	T	A	T	T	T	T	T	T	C	G	G	A	C	A	A	T	G	A	C	T	C	C	A	T

	7	7	7	7	7	7	7	7	7	7	7	8	8	8	8	8	8	8	8	8	8	8	8	8	8	8	8	8	8	8	8	8	8
	8	8	9	9	9	9	9	9	9	9	9	0	0	0	0	0	0	0	0	0	0	1	1	1	1	1	1	1	1	2	2	2	2
	8	9	1	3	4	4	7	7	8	9	9	0	1	1	2	2	4	6	6	6	6	1	1	1	3	6	6	8	9	0	1	3	3
	4	3	1	3	5	7	1	7	6	2	5	1	0	6	2	8	6	2	4	5	8	0	1	5	5	5	9	9	5	5	1	1	2
KF926377	A	C	A	T	T	A	T	G	C	C	A	T	G	C	A	C	G	A	A	A	A	C	A	A	G	G	T	T	C	C	G	T	C
MAC-T	.	.	.	.	.	.	.	.	.	.	.	.	.	.	.	.	.	C	C	T	G	.	.	.	.	.	.	.	.	.	.	.	.
Luxi	.	.	.	.	.	.	.	.	.	.	.	.	.	.	.	.	A	C	C	T	G	.	.	.	.	.	C	C	T	.	A	.	.
yak	G	T	G	A	C	G	C	A	T	T	G	C	A	T	G	T	.	C	C	T	G	A	G	G	A	A	.	C	T	T	A	C	T

	8	8	8	8	8	8	8	8	8	8	8	8	8	8	8	8	8	8	8	8	8	8	8	8	8	8	8	8	8	8	8	8	8
	2	2	2	3	3	3	3	3	3	3	3	4	4	4	4	4	4	4	4	4	4	4	5	5	5	5	5	5	5	6	6	6	6
	4	5	8	0	2	3	4	5	7	9	9	0	0	0	2	3	4	6	7	7	7	9	0	1	1	3	4	5	7	0	2	5	9
	6	0	6	9	7	3	4	3	1	5	9	4	6	7	2	7	0	7	6	7	9	5	4	5	8	0	3	1	2	8	4	7	2
KF926377	G	C	A	A	G	T	T	C	A	C	C	A	G	C	C	A	T	T	T	T	A	T	G	C	A	C	A	A	C	G	G	C
MAC-T	.	.	.	.	.	.	.	.	.	.	.	.	.	.	.	.	.	.	.	.	.	.	.	.	.	.	.	.	.	.	A	.	.
Luxi	.	.	G	G	.	.	.	.	C	.	.	.	.	.	.	.	.	C	.	.	.	G	C	A	.	.	.	.	G	.	.	.	.
yak	A	T	.	G	A	C	C	T	C	T	T	G	A	T	T	G	C	.	C	C	C	.	.	A	T	G	T	G	.	T	.	A	A

	8	8	8	8	8	8	8	8	8	8	8	8	8	8	8	8	8	9	9	9	9	9	9	9	9	9	9	9	9	9	9	9	9
	7	7	7	7	8	8	8	8	8	8	8	8	8	9	9	9	9	0	0	0	0	0	0	0	0	0	0	0	0	0	0	0	1
	2	5	7	9	0	1	3	4	6	7	7	9	9	3	3	8	9	0	2	3	3	3	6	6	6	7	7	8	9	9	9	9	2
	2	0	6	4	7	8	9	6	9	1	2	3	9	5	8	5	7	6	1	4	6	9	1	4	9	2	8	9	0	1	3	7	0
KF926377	T	A	T	C	C	A	C	A	C	C	C	T	G	T	C	T	T	C	T	T	G	T	C	A	G	A	C	T	G	A	C	C	T
MAC-T	.	.	.	.	.	.	.	.	.	.	.	.	.	.	.	.	.	.	.	.	.	.	.	.	.	.	.	.	.	.	.	.	.
Luxi	.	G	.	.	.	.	.	.	.	.	.	.	.	.	.	C	.	T	.	.	.	C	.	.	A	.	.	.	.	.	.	.	.
yak	C	.	C	T	T	G	T	G	T	T	T	C	A	C	T	.	C	T	C	C	A	C	T	G	.	G	T	C	A	G	T	T	C

	9	9	9	9	9	9	9	9	9	9	9	9	9	9	9	9	9	9	9	9	9	9	9	9	9	9	9	9	9	9	9	9	9
	1	1	1	1	1	2	2	2	2	2	2	2	3	3	3	3	3	4	4	4	4	4	4	4	5	5	5	5	5	5	5	6	6
	4	5	5	5	6	2	2	4	4	7	7	9	0	0	3	4	8	2	2	3	4	8	8	8	1	1	2	4	6	8	9	0	2
	7	1	4	6	8	2	5	3	6	0	9	7	0	3	6	5	7	1	3	2	1	0	1	9	0	9	5	6	4	2	1	3	7
KF926377	A	G	A	C	C	T	T	A	A	T	C	C	T	A	C	C	T	T	A	G	G	A	G	C	A	A	T	T	T	C	C	G	C

MAC-T	.	.	.	.	.	.	.	.	.	.	.	.	.	.	.	.	.	.	.	.	.	.	.	.	.	.	.	.	.	.	.	.	.
Luxi	.	.	G	.	.	.	.	.	G	.	.	.	.	G	.	.	.	.	.	.	.	.	A	.	.	.	.	.	.	T	.	A	.
yak	G	A	.	T	T	C	C	C	.	A	T	T	C	.	T	T	C	C	G	A	C	G	.	T	G	G	C	C	C	.	T	A	T

																					1	1	1	1	1	1	1	1	1	1	1	1	1
	9	9	9	9	9	9	9	9	9	9	9	9	9	9	9	9	9	9	9	9	0	0	0	0	0	0	0	0	0	0	0	0	0
	6	6	7	7	7	7	8	8	8	8	8	8	8	9	9	9	9	9	9	9	0	0	0	0	0	0	1	1	1	1	1	1	2
	3	5	1	6	7	7	1	3	6	6	7	8	9	1	2	2	3	4	7	9	0	2	4	4	6	7	1	2	3	3	5	5	1
	7	1	4	8	1	2	0	0	5	8	4	0	2	3	2	8	1	6	9	4	3	1	0	8	7	2	4	0	5	8	4	6	7
KF926377	T	T	C	T	C	T	T	T	C	C	C	C	A	C	A	C	A	T	T	G	C	C	C	G	G	T	C	T	T	G	C	A	A
MAC-T	.	.	.	.	.	.	.	.	.	.	.	.	.	.	.	.	.	.	.	.	.	.	.	.	.	.	.	.	.	.	.	.	.
Luxi	.	.	.	C	.	.	.	.	.	.	.	.	G	.	.	.	C	.	C	.	.	.	T	.	A	C	.	.	.	A	T	.	.
yak	C	C	T	.	T	C	C	C	T	T	T	T	.	T	G	T	.	C	C	A	T	T	T	A	A	C	G	C	C	A	.	G	G

	1	1	1	1	1	1	1	1	1	1	1	1	1	1	1	1	1	1	1	1	1	1	1	1	1	1	1	1	1	1	1	1	1
	0	0	0	0	0	0	0	0	0	0	0	0	0	0	0	0	0	0	0	0	0	0	0	0	0	0	0	0	0	0	0	0	0
	2	2	2	2	2	2	3	3	3	3	3	3	3	3	4	4	4	4	4	5	5	5	5	5	5	5	5	5	5	6	6	6	6
	5	5	6	6	7	9	0	2	3	4	5	5	7	8	1	3	4	6	9	0	1	2	4	7	7	8	8	9	9	0	0	1	2
	2	4	0	9	6	4	5	3	2	4	0	1	4	9	9	4	6	7	4	6	5	2	1	7	8	7	8	1	8	1	2	4	2
KF926377	T	C	C	A	A	C	C	C	A	A	G	C	C	C	T	C	T	T	T	T	C	T	C	G	T	A	A	A	T	G	G	G	C
MAC-T	.	.	.	.	.	.	.	.	.	.	.	.	.	.	.	.	.	.	.	.	.	.	.	.	.	.	.	.	.	.	.	.	.
Luxi	.	.	.	G	.	.	.	T	G	.	.	.	.	.	.	.	C	.	.	.	.	.	.	.	.	.	.	G	.	.	.	.	T
yak	C	T	T	.	G	T	T	T	.	G	A	T	T	T	C	T	.	C	C	C	T	C	T	A	A	G	G	.	C	A	A	A	.

	1	1	1	1	1	1	1	1	1	1	1	1	1	1	1	1	1	1	1	1	1	1	1	1	1	1	1	1	1	1	1	1	1
	0	0	0	0	0	0	0	0	0	0	0	0	0	0	0	0	0	0	0	0	0	0	0	0	0	0	0	0	0	0	0	0	0
	6	6	6	6	6	6	6	6	6	6	7	7	7	7	7	7	7	8	8	8	8	8	8	8	8	8	8	9	9	9	9	9	9
	2	3	4	5	5	6	6	7	7	9	0	1	4	5	5	7	9	1	2	2	2	3	5	6	6	8	8	1	1	3	4	5	8
	5	7	6	2	8	1	7	3	9	2	3	5	8	1	4	8	9	4	3	6	9	1	0	5	8	0	9	0	9	7	8	5	9
KF926377	T	T	C	C	C	G	C	C	T	C	C	C	C	T	A	T	C	T	G	G	C	C	T	C	G	A	T	C	C	A	C	C	C
MAC-T	.	.	.	.	.	.	.	.	.	.	.	.	.	.	.	.	.	.	.	.	.	.	.	.	.	.	.	.	.	.	T	.	.
Luxi	.	.	.	.	.	.	.	.	.	G	.	.	.	.	.	.	.	.	.	.	.	.	C	.	.	.	.	.	.	.	.	.	.
yak	A	C	T	T	T	A	T	T	C	A	T	T	T	C	G	C	T	C	A	A	T	T	C	T	A	C	C	A	T	G	.	T	T

	1	1	1	1	1	1	1	1	1	1	1	1	1	1	1	1	1	1	1	1	1	1	1	1	1	1	1	1	1	1	1	1	1
	1	1	1	1	1	1	1	1	1	1	1	1	1	1	1	1	1	1	1	1	1	1	1	1	1	1	1	1	1	1	1	1	1
	0	0	0	0	0	0	0	0	0	0	0	0	1	1	1	1	1	1	2	2	2	2	2	2	2	2	2	3	3	3	3	3	4
	0	1	3	3	5	6	6	6	7	8	8	9	0	0	3	5	5	8	0	1	1	2	3	4	4	6	8	2	3	3	8	9	0
	1	0	6	8	4	4	6	9	2	5	7	6	6	8	5	4	9	0	1	0	3	9	1	6	9	7	8	5	0	9	7	6	8
KF926377	C	T	C	C	T	T	A	G	C	G	T	T	G	C	T	C	T	T	C	C	C	G	T	G	G	C	G	C	A	T	C	C	C
MAC-T	.	.	.	.	.	.	.	.	.	.	.	.	.	.	.	.	.	.	.	.	.	.	.	.	.	.	.	.	.	.	.	.	.
Luxi	T	.	T	.	.	.	.	A	.	.	.	.	.	.	C	.	.	.	T	.	.	.	.	.	.	T	.	.	G	.	.	.	T
yak	T	C	.	T	C	C	C	A	T	A	G	C	A	T	C	T	C	C	.	T	T	A	C	A	A	.	A	T	.	C	T	T	.

	1	1	1	1	1	1	1	1	1	1	1	1	1	1	1	1	1	1	1	1	1	1	1	1	1	1	1	1	1	1	1	1	1
	1	1	1	1	1	1	1	1	1	1	1	1	1	1	1	1	1	1	1	1	1	1	1	1	1	1	1	1	1	1	1	1	1
	4	4	4	4	4	4	4	4	5	5	6	6	6	6	7	7	7	7	7	7	7	7	7	8	8	8	8	8	8	8	8	8	8
	2	2	2	4	5	7	7	8	5	8	2	3	3	7	1	2	4	5	5	6	8	8	9	0	1	2	4	4	4	5	5	5	6
	0	3	9	1	3	1	4	6	2	2	8	3	4	3	1	1	7	6	9	5	3	6	2	4	0	5	3	4	6	2	3	8	8
KF926377	T	C	C	G	C	C	T	C	C	C	T	T	C	G	T	G	T	C	G	T	A	T	C	T	G	G	A	T	A	C	C	C	C

MAC-T	.	.	.	.	.	.	.	.	.	.	.	.	.	.	.	.	.	.	.	.	.	.	.	.	.	.	.	.	.	.	.	.	.
Luxi	C	.	.	.	.	.	.	.	.	.	.	.	.	.	.	.	.	.	.	.	.	.	.	C	.	.	G	.	.	.	.	.	.
yak	C	T	T	A	T	T	C	T	T	T	C	C	T	A	C	A	C	T	A	C	G	C	T	.	A	A	G	C	G	T	T	T	T

	1	1	1	1	1	1	1	1	1	1	1	1	1	1	1	1	1	1	1	1	1	1	1	1	1	1	1	1	1	1	1	1	1
	1	1	2	2	2	2	2	2	2	2	2	2	2	2	2	2	2	2	2	2	2	2	2	2	2	2	2	2	2	2	2	2	2
	8	8	0	0	0	0	0	0	1	1	1	1	1	1	1	1	1	1	2	2	2	2	2	2	2	3	3	3	3	3	3	3	3
	7	7	1	1	2	3	5	8	2	3	4	4	5	5	7	7	7	9	0	2	2	3	7	7	9	1	1	2	3	3	4	7	7
	1	9	7	8	2	6	9	0	8	6	5	9	7	8	1	4	9	4	8	4	9	5	4	5	3	4	9	8	1	5	0	6	7
KF926377	A	C	T	C	T	C	T	A	C	A	A	T	T	A	T	T	T	C	A	G	C	C	C	C	A	C	C	T	T	T	C	T	A
MAC-T	.	.	.	.	.	.	.	.	.	.	.	.	.	.	.	.	.	.	.	.	.	.	.	.	.	.	.	.	.	.	.	.	.
Luxi	.	.	.	.	.	.	.	.	.	G	.	.	.	.	.	.	C	.	.	.	.	T	.	.	.	.	.	.	.	.	.	.	.
yak	T	T	C	T	C	T	C	G	T	.	G	C	A	G	C	C	C	T	G	A	T	T	T	T	G	T	T	C	C	C	T	C	G

	1	1	1	1	1	1	1	1	1	1	1	1	1	1	1	1	1	1	1	1	1	1	1	1	1	1	1	1	1	1	1	1	1
	2	2	2	2	2	2	2	2	2	2	2	2	2	2	2	2	2	2	2	2	2	2	2	2	2	2	2	2	2	2	2	2	2
	3	4	4	4	4	4	4	4	4	5	5	5	5	5	5	5	5	5	6	6	6	6	6	6	6	6	6	6	6	7	7	7	7
	7	3	3	6	7	8	9	9	9	1	2	2	2	4	5	8	8	9	0	1	2	2	5	5	5	7	7	8	8	0	2	2	3
	8	0	4	9	0	7	3	4	6	4	0	6	9	7	6	0	3	8	4	3	3	5	5	8	9	3	6	5	8	0	2	9	1
KF926377	T	T	T	T	C	T	G	C	C	C	C	G	A	C	C	A	A	T	C	C	A	C	C	T	T	G	C	T	C	C	A	G	G
MAC-T	.	.	.	.	.	.	.	.	.	.	.	.	.	.	.	.	.	.	.	.	.	.	.	.	.	.	.	.	.	.	.	.	.
Luxi	C	.	C	C	T	.	.	.	.	T	.	.	.	.	.	.	.	.	.	.	G	.	.	.	.	A	T	C	.	.	.	.	.
yak	.	C	C	C	T	C	A	A	A	T	A	A	G	T	T	G	G	C	T	T	G	T	T	C	C	A	T	C	A	T	G	A	A

	1	1	1	1	1	1	1	1	1	1	1	1	1	1	1	1	1	1	1	1	1	1	1	1	1	1	1	1	1	1	1	1	1
	2	2	2	2	2	2	2	2	2	2	2	2	2	2	2	2	2	2	2	2	2	2	2	2	2	2	3	3	3	3	3	3	3
	7	7	7	7	7	7	8	8	8	8	8	8	8	9	9	9	9	9	9	9	9	9	9	9	9	9	0	0	0	0	0	0	0
	3	3	4	5	5	6	0	5	6	6	8	9	9	0	0	1	2	2	2	2	4	4	6	7	7	8	0	3	5	5	7	8	8
	3	9	1	1	9	1	2	0	5	8	0	5	8	1	4	6	3	4	5	8	6	7	5	0	6	5	6	0	1	7	5	4	7
KF926377	C	C	T	T	C	T	G	C	A	G	C	T	C	T	C	C	T	A	C	C	C	T	C	T	A	C	A	C	A	T	T	T	C
MAC-T	.	.	.	.	.	.	.	.	.	.	.	.	.	.	.	.	.	.	.	.	.	.	.	.	.	.	.	.	.	.	.	.	.
Luxi	T	.	.	C	.	.	A	.	.	.	.	.	.	C	.	.	.	T	T	.	.	.	.	.	.	.	G	.	.	A	.	.	.
yak	.	T	C	C	T	C	A	T	G	A	T	C	T	C	A	T	C	.	.	T	T	C	T	C	G	T	G	T	G	A	C	A	T

	1	1	1	1	1	1	1	1	1	1	1	1	1	1	1	1	1	1	1	1	1	1	1	1	1	1	1	1	1	1	1	1	1
	3	3	3	3	3	3	3	3	3	3	3	3	3	3	3	3	3	3	3	3	3	3	3	3	3	3	3	3	3	3	3	3	3
	0	1	1	1	1	1	1	1	1	2	2	2	2	3	3	3	3	3	3	3	3	3	4	4	4	4	4	4	4	4	4	4	4
	9	0	0	2	4	4	7	7	8	0	1	5	7	3	5	6	7	7	8	8	9	9	0	2	2	3	3	3	3	5	5	8	8
	9	2	5	9	4	7	4	7	9	7	6	6	6	7	1	9	2	5	1	7	0	3	2	0	1	0	4	5	8	6	9	0	6
KF926377	T	C	C	C	C	T	T	T	A	G	C	A	C	C	T	T	T	C	C	T	T	T	T	C	C	G	A	T	T	T	G	A	T
MAC-T	.	.	.	.	.	.	.	.	.	.	.	.	.	.	.	.	.	.	.	.	.	.	.	.	.	.	.	.	.	.	.	.	.
Luxi	C	A	T	.	.	.	.	.	G	.	.	.	T	.	.	.	C	.	T	.	.	.	.	.	.	.	G	.	C	.	.	.	.
yak	C	.	.	T	T	A	C	C	.	A	T	G	T	T	C	C	.	T	.	C	C	C	C	T	T	A	.	C	C	C	A	G	C

	1	1	1	1	1	1	1	1	1	1	1	1	1	1	1	1	1	1	1	1	1	1	1	1	1	1	1	1	1	1	1	1	1
	3	3	3	3	3	3	3	3	3	3	3	3	3	3	3	3	3	3	3	3	3	3	3	3	3	3	3	3	3	3	3	3	3
	5	5	5	5	5	5	5	5	5	5	5	5	6	6	6	6	6	6	6	6	6	6	6	6	7	7	8	8	8	8	8	8	8
	0	0	1	1	2	2	3	4	5	6	6	8	0	2	2	2	2	3	4	7	7	9	9	9	3	9	0	1	4	5	7	8	8
	4	7	0	3	2	8	8	0	5	4	5	5	5	1	4	7	9	9	7	2	8	0	3	9	3	2	7	6	0	8	9	3	8
KF926377	A	C	C	T	T	T	A	T	G	C	C	T	T	C	T	T	T	T	A	G	A	G	T	C	C	C	C	C	C	G	C	T	C

MAC-T	.	.	.	.	.	.	.	.	.	.	.	.	.	.	.	.	.	.	.	.	.	.	.	.	T	.	.	.	.	.	.	.	.
Luxi	.	.	.	.	.	.	.	.	A	.	T	C	.	.	.	.	C	.	.	.	G	A	C	.	T	.	.	.	.	.	.	C	.
yak	G	T	T	C	C	C	G	C	A	T	.	C	C	T	C	C	.	C	T	A	C	A	C	T	.	T	T	T	T	A	T	C	T

	1	1	1	1	1	1	1	1	1	1	1	1	1	1	1	1	1	1	1	1	1	1	1	1	1	1	1	1	1	1	1	1	1
	3	3	3	3	3	3	3	4	4	4	4	4	4	4	4	4	4	4	4	4	4	4	4	4	4	4	4	4	4	4	4	4	4
	8	8	9	9	9	9	9	0	0	0	0	0	0	0	1	1	1	1	1	1	1	1	1	2	2	2	2	2	2	2	2	2	3
	9	9	0	0	1	5	8	1	2	3	5	6	6	9	0	0	0	2	3	3	6	9	9	0	1	4	5	7	8	8	9	9	1
	4	7	0	9	0	6	9	0	8	7	2	4	7	9	3	6	9	1	0	9	2	3	9	5	4	4	6	4	0	8	2	5	0
KF926377	C	T	C	C	T	T	G	C	T	G	T	C	C	C	A	T	T	T	G	G	C	C	T	A	T	T	T	G	C	A	G	T	T
MAC-T	.	.	.	.	.	.	.	.	.	.	.	.	.	.	.	.	.	.	.	.	.	.	.	.	.	.	.	.	.	.	.	.	.
Luxi	.	.	.	A	C	.	.	.	.	A	.	.	T	.	.	.	.	C	A	A	.	.	.	.	.	.	C	.	.	.	.	.	.
yak	T	C	T	.	C	A	A	T	C	A	C	T	.	T	C	C	C	C	A	A	T	T	C	G	C	C	C	A	T	G	A	C	C

	1	1	1	1	1	1	1	1	1	1	1	1	1	1	1	1	1	1	1	1	1	1	1	1	1	1	1	1	1	1	1	1	1
	4	4	4	4	4	4	4	4	4	4	4	4	4	4	4	4	4	4	4	4	4	4	4	4	4	4	4	4	4	4	4	4	4
	3	3	3	3	3	3	4	4	4	4	4	5	5	5	5	5	5	5	6	6	6	6	6	6	6	6	6	7	7	7	7	7	7
	1	2	4	8	9	9	0	0	1	1	6	0	2	3	3	6	7	8	0	0	1	1	2	2	3	3	8	0	1	4	5	8	8
	6	2	9	8	1	7	3	6	2	7	9	4	3	2	8	2	1	3	4	7	0	6	5	9	1	5	8	9	3	8	7	1	4
KF926377	T	G	T	C	A	T	G	A	G	G	T	C	C	G	C	T	C	C	A	A	T	T	G	A	C	C	C	T	A	C	C	G	T
MAC-T	.	.	.	.	.	.	.	.	.	.	.	.	.	.	.	.	.	.	.	.	.	.	.	.	.	.	.	.	.	.	.	.	.
Luxi	C	.	.	.	.	.	.	.	A	A	.	T	.	.	.	.	.	.	.	G	.	.	.	.	.	.	.	.	.	.	.	.	.
yak	.	A	C	T	G	A	A	G	A	A	C	.	T	A	T	C	T	T	G	.	C	C	A	G	A	T	T	C	G	T	T	A	C

	1	1	1	1	1	1	1	1	1	1	1	1	1	1	1	1	1	1	1	1	1	1	1	1	1	1	1	1	1	1	1	1	1
	4	4	4	4	4	4	4	4	4	4	4	4	4	4	4	4	4	4	5	5	5	5	5	5	5	5	5	5	5	5	5	5	5
	8	8	8	8	8	8	8	8	8	8	8	8	9	9	9	9	9	9	0	0	0	0	0	0	0	0	0	0	1	1	1	1	1
	0	1	1	2	3	4	5	6	7	7	8	9	1	3	4	5	6	7	0	0	1	1	3	6	6	7	8	8	0	3	4	5	6
	2	4	8	6	8	1	9	8	4	7	9	8	0	4	0	2	2	3	3	6	2	5	9	0	9	8	1	3	6	5	7	8	8
KF926377	G	A	T	C	T	T	T	C	G	C	C	T	C	C	A	C	T	C	A	C	C	A	T	T	T	C	C	T	C	C	T	T	C
MAC-T	.	.	.	.	.	.	.	.	.	.	.	.	.	.	.	.	.	.	.	.	.	.	.	.	.	.	.	.	.	.	.	.	.
Luxi	.	.	.	T	.	.	C	.	.	.	.	C	.	.	.	.	.	.	.	.	.	.	.	.	.	.	.	.	T	T	C	.	.
yak	A	G	C	.	C	C	.	T	A	T	T	C	A	T	G	T	C	T	G	T	T	G	C	C	C	T	T	C	.	T	C	C	T

	1	1	1	1	1	1	1	1	1	1	1	1	1	1	1	1	1	1	1	1	1	1	1	1	1	1	1	1	1	1	1	1	1
	5	5	5	5	5	5	5	5	5	5	5	5	5	5	5	5	5	5	5	5	5	5	5	5	5	5	5	5	5	5	5	5	5
	1	1	2	2	2	2	2	2	2	2	2	2	3	3	3	3	3	3	3	4	4	4	4	4	4	4	4	5	5	5	5	5	5
	7	9	0	1	1	2	3	4	5	7	8	9	0	1	2	5	7	8	9	1	2	2	3	4	6	9	9	1	2	5	5	6	6
	4	5	7	1	3	8	7	0	0	6	8	1	9	2	7	4	2	7	9	4	3	6	5	1	2	3	5	3	8	5	8	0	4
KF926377	C	G	G	C	C	T	A	A	G	T	A	C	C	T	G	C	C	A	C	T	A	C	A	C	A	G	C	G	T	T	C	C	C
MAC-T	.	.	.	.	.	.	.	.	.	.	.	.	.	.	.	.	.	.	.	.	.	.	.	.	.	.	.	.	.	.	.	.	.
Luxi	.	.	.	.	.	.	.	.	.	.	G	.	T	.	A	.	.	.	.	.	.	.	.	.	.	.	.	.	.	.	.	.	T
yak	T	A	A	T	A	C	T	G	A	C	.	A	T	C	A	T	T	G	T	C	T	T	G	T	C	A	T	A	C	C	T	T	T

	1	1	1	1	1	1	1	1	1	1	1	1	1	1	1	1	1	1	1	1	1	1	1	1	1	1	1	1	1	1	1	1	1
	5	5	5	5	5	5	5	5	5	5	5	5	5	5	5	5	5	5	5	5	5	5	5	5	5	5	5	5	5	5	5	5	5
	5	5	5	5	5	6	6	6	6	6	6	6	6	7	7	7	7	7	8	8	8	8	8	8	8	8	8	8	8	9	9	9	9
	8	8	8	9	9	0	1	2	3	4	5	7	9	1	4	4	5	9	1	2	3	3	4	4	5	7	7	7	8	1	1	1	1
	0	2	3	1	4	6	8	8	0	0	7	9	9	2	1	2	2	6	9	0	0	2	7	8	5	2	4	9	2	6	7	8	9
KF926377	G	C	C	T	C	G	G	A	C	T	G	T	G	T	G	C	A	A	A	C	T	T	T	C	C	G	A	C	C	T	A	A	C

MAC-T	.	.	.	.	.	.	.	.	.	.	A	.	.	.	.	.	.	.	.	.	.	.	.	.	.	.	.	.	.	.	.	.	.
Luxi	A	.	.	.	T	A	A	G	T	.	.	.	.	.	.	T	G	.	G	.	.	.	.	.	.	.	.	.	.	.	.	.	.
yak	A	T	A	C	T	A	A	.	T	C	A	C	A	C	A	.	G	G	G	T	C	C	C	T	T	A	C	T	T	-	-	-	-

	1	1	1	1	1	1	1	1	1	1	1	1	1	1	1	1	1	1	1	1	1	1	1	1	1	1	1	1	1	1	1	1	1
	5	5	5	5	5	5	5	5	5	5	5	5	5	5	5	5	5	5	5	5	5	5	5	5	5	5	5	5	6	6	6	6	6
	9	9	9	9	9	9	9	9	9	9	9	9	9	9	9	9	9	9	9	9	9	9	9	9	9	9	9	9	0	0	0	0	0
	2	2	2	2	2	2	2	3	3	3	3	4	4	4	5	5	5	5	5	5	6	6	6	6	6	7	9	9	2	4	5	5	5
	0	1	2	3	4	5	7	3	4	7	8	0	5	9	0	2	4	5	7	8	0	2	3	4	6	5	5	6	3	8	0	1	2
KF926377	A	C	G	C	C	C	T	G	A	A	C	G	A	A	C	T	C	G	A	A	G	G	T	A	T	C	A	A	G	G	C	C	C
MAC-T	.	.	.	.	.	.	.	.	.	.	.	.	.	.	.	.	.	.	.	.	.	.	.	.	.	.	.	.	.	.	.	.	T
Luxi	.	.	.	.	.	.	.	.	.	.	.	.	.	.	.	C	G	.	.	.	A	.	.	.	.	.	G	.	A	.	T	.	T
yak	-	-	-	-	-	-	C	T	C	C	A	A	C	G	T	C	A	A	G	G	.	A	A	T	C	T	.	T	.	A	T	T	.

	1	1	1	1	1	1	1	1	1	1	1	1	1	1	1	1	1	1	1	1	1	1	1	1	1	1	1	1	1	1	1	1	1
	6	6	6	6	6	6	6	6	6	6	6	6	6	6	6	6	6	6	6	6	6	6	6	6	6	6	6	6	6	6	6	6	6
	0	0	0	0	0	0	0	0	0	0	0	0	0	0	1	1	1	1	1	1	1	1	1	1	1	1	1	1	1	1	1	1	1
	5	5	5	6	6	6	7	7	8	8	8	9	9	9	0	0	1	1	1	1	1	1	1	2	2	2	2	2	2	2	2	3	3
	7	8	9	2	3	8	4	5	3	5	6	4	5	7	3	9	0	1	3	4	6	7	8	0	2	3	5	6	7	8	9	0	1
KF926377	A	G	C	C	A	A	A	T	G	C	T	G	T	C	G	T	T	C	T	T	T	T	G	T	G	T	T	A	T	C	T	G	T
MAC-T	.	.	.	T	.	.	G	.	.	.	.	.	.	.	.	.	.	.	.	.	.	.	.	.	.	.	.	.	.	.	.	A	.
Luxi	.	A	T	T	.	.	.	C	A	T	.	.	.	.	A	.	C	.	.	C	.	C	A	C	A	C	.	.	.	.	.	A	C
yak	G	.	T	T	G	G	G	A	A	T	C	A	C	T	.	C	C	T	C	.	C	.	A	C	A	C	-	-	-	-	-	-	-

	1	1	1	1	1	1	1	1	1	1	1	1	1	1	1	1	1	1	1	1	1	1	1	1
	6	6	6	6	6	6	6	6	6	6	6	6	6	6	6	6	6	6	6	6	6	6	6	6
	1	1	1	1	1	1	1	1	2	2	2	2	2	2	2	2	2	2	2	2	3	3	3	3
	3	3	3	4	4	4	6	9	0	0	0	2	3	3	4	4	5	6	6	9	0	0	0	0
	3	8	9	2	4	8	5	7	0	5	6	9	0	3	8	9	6	1	5	5	1	2	3	9
KF926377	A	T	T	T	A	T	T	G	A	T	C	A	A	C	C	C	T	C	G	T	A	C	G	T
MAC-T	.	.	.	.	A	.	.	.	.	.	.	.	.	.	.	.	.	.	.	.	.	.	.	.
Luxi	.	C	C	.	-	C	.	A	.	.	.	.	G	.	T	T	.	.	.	.	G	T	.	.
yak	G	C	C	C	-	C	C	.	G	C	T	G	.	T	T	T	C	T	A	C	.	.	C	C

Besides SNPs, yak has a T insert at the position 1172 (in 12S), G at 1553 and A at 1855 (in 16S), T at 5506 (in tRNA-Asn), T at 16056 and A at 16202 (in CR); Luxi has an A insert at the position 16202 (in CR); position is referenced to the sequence of KF926377.

Table S2 The score for amino acid change among three bovine mitogenomes

Gene	Position	MAC-T	Luxi	yak	Grantham Score
ND1	29	Gly	Ala	Gly	60
	68	Ala	Ala	Thr	58
	77	Met	Met	Leu	15
	93	Tyr	Tyr	His	83
	167	Thr	Ile	Ile	89
	172	Met	Met	Thr	81
	236	Ile	Ile	Met	10
	260	Thr	Thr	Ile	89
	269	Ser	Ser	Leu	145
ND2	7	Ile	Ile	Thr	89
	8	Ile	Ile	Thr	89
	159	Val	Val	Ile	29
	210	Ile	Ile	Thr	89
	242	Val	Val	Ile	29
	301	Thr	Thr	Ala	58
	336	Met	Met	Thr	81
COI	453	Ile	Ile	Val	29
COII	99	Thr	Thr	Ala	58
	187	Ser	Ser	Thr	58
ATP8	28	Val	Ile	Ile	29
ATP6	35	His	His	Tyr	83
	41	Pro	Pro	Ser	74
	53	Thr	Ala	Thr	58
COIII	32	Thr	Thr	Ala	58
	40	Met	Met	Thr	81
	41	Thr	Thr	Ala	58
	61	Val	Val	Ile	29
	62	Ile	Val	Ile	29
	157	Lys	Lys	Asn	94
	171	Val	Ile	Val	29
ND3	82	Ala	Thr	Thr	58
ND4L	5	Tyr	Tyr	His	83
	13	Thr	Thr	Ala	58
ND4	17	Leu	Leu	Met	15
	20	Asn	Asn	Gly	80
	21	Asn	Ser	Asn	46
	25	Val	Val	Ile	29
	29	Ala	Ala	Thr	58
	55	Leu	Val	Met	Leu32Val/Leu15Met/Val21Met
	101	Ser	Ser	Leu	145
	117	Leu	Leu	Phe	22
	140	Met	Thr	Thr	81
	170	Thr	Thr	Met	81
	186	Val	Val	Met	21
	193	Val	Val	Ile	29

	234	Val	Val	Ile	29
	382	Val	Val	Met	21
	398	Val	Val	Met	21
	448	Thr	Thr	Ser	58
ND5	7	Leu	Leu	Phe	22
	17	Met	Met	Val	21
	21	Met	Met	Thr	81
	22	Met	Met	Thr	81
	24	Phe	Leu	Leu	22
	29	Pro	Pro	Ser	74
	39	Ala	Ala	Thr	58
	56	His	His	Tyr	83
	62	Ile	Ile	Val	29
	90	Ile	Thr	Val	Ile89Thr/Ile29Val/Thr69Val
	109	Tyr	His	His	83
	129	Leu	Leu	Met	15
	172	Ile	Val	Val	29
	205	Asn	Asn	Asp	23
	207	Ser	Ser	Asn	46
	208	Asp	Asp	Asn	23
	211	Met	Met	Thr	81
	217	Ala	Ala	Val	64
	272	Tyr	Phe	His	Tyr22Phe/Tyr83His/Phe100His
	346	Ile	Ile	Met	10
	383	Met	Met	Val	21
	438	Pro	Pro	Ser	74
	441	Val	Val	Ile	29
	442	Asn	Ser	Asn	46
	477	Ile	Ile	Val	29
	499	Ile	Ile	Thr	89
	507	Met	Thr	Met	81
	513	Tyr	Tyr	Phe	22
	600	Ile	Met	Ile	10
ND6	15	Met	Met	Ile	10
	94	Val	Val	Ile	29
	115	Val	Val	Ile	29
	162	Leu	Leu	Phe	22
Cytb	39	Ile	Ile	Val	29
	67	Thr	Thr	Ala	58
	190	Met	Met	Thr	81
	215	Val	Val	Ala	64
	246	Ala	Ala	Thr	58
	316	Met	Met	Ile	10
	327	Ala	Ala	Thr	58
	349	Thr	Thr	Ile	89
	356	Val	Ile	Ile	29
	357	Leu	Leu	Met	15
	372	Ile	Val	Ile	29

Table S3 Primer information for bovine mitochondrial genome sequencing.

Primer Pair No.	Primer sequences 5'-3'	Position*	Expectant size
1	F: GGTCATACGATTAACCCAAG	682-701	1140
	R: TGGACAACCAGCTATCACCA	1802-1821	
2	F: CGAAACCAGACGAGCTACT	1698-1716	1343
	R: GCTCTGCCACCTTAACTAAG	3021-3040	
3	F: ACGTGATCTGAGTTCAGACC	2854-2873	1191
	R: GGATGCCTGATGTAAGGATG	4025-4044	
4	F: CCTATGAATCCGAGCATCCT	3911-3930	1434
	R: CTTTGAAGGCTCTTGGTCTG	5325-5344	
5	F: CTCTCCTATCCATAGGAGGA	5006-5025	1179
	R: GTAATGAAGTTGATGGCTCC	6165-6184	
6	F: GCATCCTCTATAGTTGAAGC	6027-6046	1246
	R: GGCTTGAAACCAATAGTAGG	7253-7272	
7	F: CTCGACGATACTCCGACTA	6997-7015	1165
	R: GTCAGTCATGTTGACGTGTC	8142-8161	
8	F: GAGGACTTAAGCTTCGACTC	7714-7733	1245
	R: TGCAGATATAGGCTGACTAG	8939-8958	
9	F: CAATAAACCTAGGCATAGCC	8586-8605	1224
	R: CTAGACCGAAACTAGCTGAT	9790-9809	
10	F: ACTTCTAACCACCACTTCGG	9652-9671	1528
	R: GGTAGTCAAAGGTGGAGGC	11161-11179	
11	F: GCTAGCCAACATCATCTATC	10764-10783	1249
	R: GGAGCATAGAATTAGCAGTTC	11992-12012	
12	F: ATCTCGCCTTCCTTTACACG	11805-11824	1225
	R: GATGTGGAGAAGGCGATGAT	13010-13029	
13	F: ACTTCCCTCTGCAATAGAAG	12805-12824	1178
	R: GTTATTGTAACTGGGTGGTC	13963-13982	
14	F: ATCAGCATCCTCCCTTCTAG	13750-13769	1287
	R: GGTTGCTTTGTCTACTGAGA	15017-15036	
15	F: ATACACGCAAACGGAGCTTC	14758-14777	1288
	R: GTACTTGCTTATATGCATGGG	16025-16045	
16	F: TCCCTAAGACTCAAGGAAGA	15717-15736	822
	R: ATGTCCTGTGACCATTGACT	180-199	
17	F: AAACCAGCAACCCGCTAG	16180-16197	574
	R: GACTCATCTAGGCATTTTCAGTG	392-414	
18	F: AACTGCATCTTGAGCACCAG	136-155	880
	R: TGAGGTTTATCGGGGTTTATC	995-1015	

*Positions referred to KF926377.1.

Table S4 Sequences of the primers used for quantification of target and reference genes.

Gene	Primer sequences 5'-3'	Accession no.	Source
PPARGC1A	F: GAAGGCAATTGAAGAGCGC<br>R: TCGACCTGCGCAAAGTGTAT	NM_177945.3	[1]
TFAM	F: ATGCTTACAGGGCAGACTGG<br>R: AGCTTTACCTGTGATGTGCCA	NM_001034016.2	[1]
NRF1	F: CCCAAACTGAGCACATGG<br>R: GTTAAGTATGTCTGAATCGTC	NM_001098002.2	[1]
NRF2	F: TCCAACCTTTGTCGTCATCA<br>R: TTGCCCGTAGCTCATCTCTT	AB162435	[2]
GPAM	F: GCAGGTTTATCCAGTATGGCATT<br>R: GGACTGATATCTTCCTGATCATCTTG	AY515690	[3]
ACSL1	F: GTGGGCTCCTTTGAAGAACTGT<br>R: ATAGATGCCTTTGACCTGTTCAAAT	BC119914	[3]
GAPDH	F: AGATGGTGAAGGTCGGAGTG<br>R: CGTTCTCTGCCTTGACTGTG	NM_001034034.2	[4]

References for Supplementary Materials

1 Laubenthal, L. *et al.* Mitochondrial DNA copy number and biogenesis in different tissues of early-and late-lactating dairy cows. *J. Dairy Sci.* **99**, 1571-1583 (2015).

2 Mastromonaco, G. F., Favetta, L. A., Smith, L. C., Filion, F. & King, W. A. The influence of nuclear content on developmental competence of gaur x cattle hybrid in vitro fertilized and somatic cell nuclear transfer embryos. *Biol Reprod* **76**, 514-523 (2007).

3 Bionaz, M. & Loor, J. J. Gene networks driving bovine milk fat synthesis during the lactation cycle. *BMC Genomics* **9**, 366-366 (2008).

4 Cui, X. *et al.* Transcriptional profiling of mammary gland in Holstein cows with extremely different milk protein and fat percentage using RNA sequencing. *BMC Genomics* **15**, 1-15 (2014).
